# Phylogenetic analysis of porcine parvoviruses from swine samples in China

**DOI:** 10.1186/1743-422X-8-320

**Published:** 2011-06-26

**Authors:** Xiaofang Hao, Zengjun Lu, Pu Sun, Yuanfang Fu, Yimei Cao, Pinghua Li, Xingwen Bai, Huifang Bao, Baoxia Xie, Yingli Chen, Dong Li, Zaixin Liu

**Affiliations:** 1 State Key Laboratory of Veterinary Etiological Biology, Key Laboratory of Animal Virology of Ministry of Agriculture, Lanzhou Veterinary Research Institute, Chinese Academy of Agriculture Sciences, Lanzhou, Gansu 730046, PR China

## Abstract

**Background:**

Porcine parvovirus (PPV) usually causes reproductive failure in sows. The objective of the present study was to analyze the phylogenetic distribution and perform molecular characterization of PPVs isolated in China, as well as to identify two field strains, LZ and JY. The data used in this study contained the available sequences for NS1 and VP2 from GenBank, as well as the two aforementioned Chinese strains.

**Results:**

Phylogenetic analysis shows that the PPV sequences are divided into four groups. The early Chinese PPV isolates are Group I viruses, and nearly all of the later Chinese PPV isolates are Group II viruses. LZ belongs to group II, whereas the JY strain is a Group III virus. This is the first report on the isolation of a Group III virus in China. The detection of selective pressures on the PPV genome shows that the NS1 and VP2 genes are under purifying selection and positive selection, respectively. Moreover, the amino acids in the VP2 capsid are highly variable because of the positive selection.

**Conclusions:**

Our study provides new molecular data on PPV strains in China, and emphasizes the importance of etiological studies of PPV in pigs.

## Background

From a worldwide perspective, the porcine parvovirus (PPV) is one of the most common viral causes of porcine reproductive failure. PPV is of the genus Parvovirus, a group of viruses of that also infect cattle, cats, dogs, geese, rats, mice, mink, and raccoons. Although PPV is distinguishable from parvoviruses of all other species, it is antigenically related to other parvoviruses such as the canine parvovirus (CPV) and the feline panleukopenia virus (FPV) [1]. The PPV genome is a single strand DNA with a terminal palindromic structure. Its size is about 5 kb. The PPV particle is composed of three viral polypeptides, VP1, VP2, and VP3, with molecular weights of 83, 64, and 62 kDa, respectively. In vitro expression of the VP2 gene may spontaneously form the capsid. The structure of the VP2 capsid was resolved using X-ray crystallography and was found to be similar to the CPV, FPV, and minute viruses of mice (MVM) [2]. Pigs immunized with these virus-like particles mounted an immune response identical to that toward commercial vaccines [3]. Recently, the genome of PPV was found to contain a small open reading frame, designated as SAT, with a start codon downstream of the VP2 initiation codon [4]. In addition to these capsid proteins, some nonstructural polypeptides, such as NS1, NS2, and NS3, have also been identified in PPV-infected cells. Differentiation of infected pigs from vaccinated ones based on antibodies for NS1 protein using inactivated vaccine is possible, as NS1 protein is absent in purified virions used as a killed vaccine [5,6].

Since the first discovery of PPV in 1983, the virus has spread widely in swine in China. Pigs are continuously threatened by PPV. Many new field strains were isolated from pigs in China, such as the China strain [6] and the BQ and ZJ strains [7]. Although inactivated and attenuated vaccines are widely used in swine in China, PPV infection is still a serious infectious disease. Therefore, porcine parvovirus surveillance in China is needed to learn the prevalence of PPV infections and to provide efficient epidemiological data for its control.

## Results

### Genomic sequences of the LZ and JY strains

The entire coding sequences of the LZ ad JY isolates contain 4509 and 4485 nucleotides, respectively, with no insertions or deletions in the coding regions. The sequences behind the VP2 stop codon of the LZ and JY isolates lack 105 and 127 nucleotides, respectively. This absence was also detected in some German isolates (e.g., PPV strains 15a, 143a, 106b, and Tornau), the American isolate Kresse, and some Chinese isolates (e.g., PPV strains BQ and ZJ). The complete sequences of LZ and JY strains were submitted to GenBank [GenBank: HM627652 and HM627653].

### Phylogenetic analysis of porcine parvoviruses isolated in China

**Figure 1:**
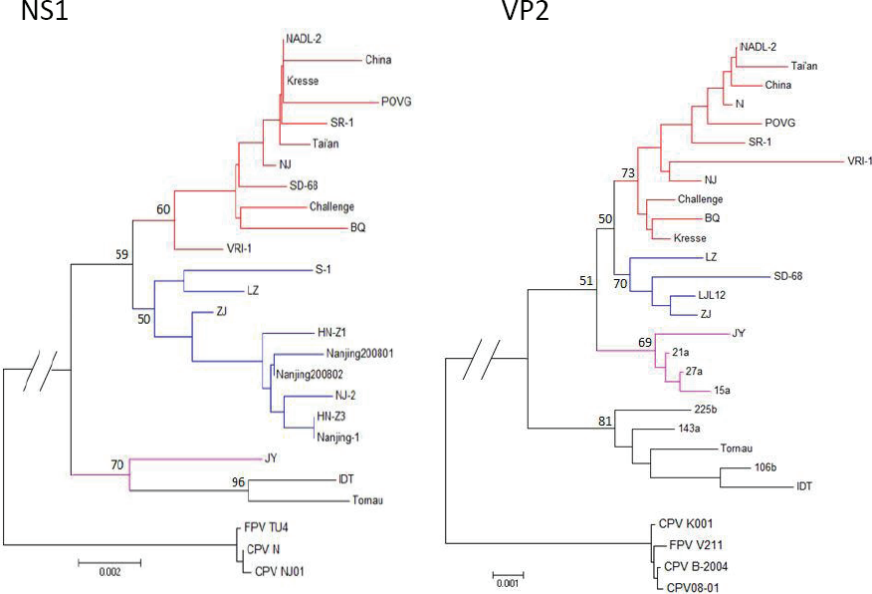
**Phylogenetic trees based on the neighbor-joining method for the 23 NS1 and 24 VP2 sequences**. The tree was constructed using MEGA version 4.1. Bootstrap values obtained from 1000 replicates are shown at the major nodes. The NS1 and VP2 sequences of CPV and FPV were included as outgroups. The different groups in the trees are marked by different colors.

To characterize the genetic relationships of porcine parvoviruses isolated in China, and to identify the two PPV strains in this study, the phylogenetic trees of NS1 and VP2 nucleotide sequences were constructed based on the neighbor-joining method (Figure 1).

Four main groups or clades were formed in the NS1 and VP2 phylogenetic trees despite the fact that some isolates were not provided in the NS1 tree. Group I comprises some American strains (e.g., NADL-2, POVG, and Kresse), Chinese strains (e.g., China, SR-1, Tai'an, BQ, NJ, and N), the UK strain Challenge, and the Korean strain VRI-1. Group II contains the Chinese strains LZ and ZJ, some strains in the NS1 dataset (S-1, HN-Z1, Nanjing200801, Nanjng200802, NJ-2, HN-Z3, and Nanjing-1), and one strain in the VP2 dataset (LJL12). The strain SD-68 is placed in Group I in the NS1 tree, and under Group II in the VP2 tree. Group III contains three German isolates (21a, 27a, and 15a) and our isolate JY. Finally, Group IV is formed by five German isolates 225b, 143a, Tornau, 106b, and IDT. The Chinese isolates are mainly in Group I and II. Our isolate LZ belongs to Group II. However, another isolate, JY, was characterized as a Group III virus. This is the first isolation of a Group III virus in China.

### Selective pressure analysis of the PPV genome

**Figure 2:**
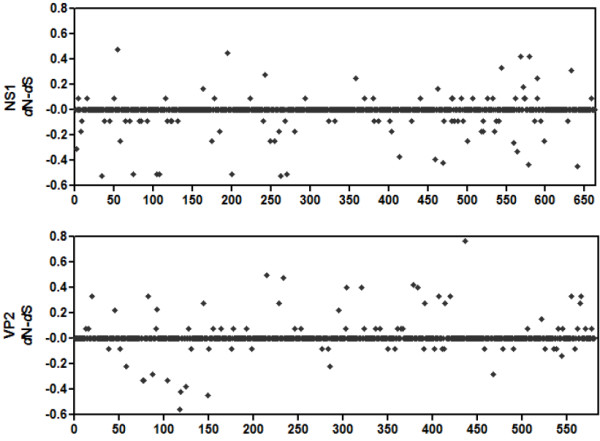
**Differences between non-synonymous and synonymous substitutions (dN - dS) for the NS1 and VP2 genes**. Numbers on the horizontal axis represent amino acid positions.

The selective pressures on the PPV genome were assessed by calculating the difference between non-synonymous (dN) and synonymous (dS) rates for the NS1 and VP2 genes. The average difference between dN and dS substitution rates (dN - dS) for NS1 was clearly negative (-0.009955 ± 0.003446), based on the SNAP web utility http://hcv.lanl.gov/content/sequence/SNAP/SNAP.html results. This indicates that NS1 is under purifying selection. In contrast, the average dN - dS for VP2 is clearly positive (0.006535 ± 0.003717), suggesting a positive selection pattern (Figure 2).

The distribution of dN - dS along the NS1 sequence shows that the negative values are distributed among the first 130 amino acids and around residue 550. According to previous studies, some amino acid residues of VP2 protein, such as 378, 383, 436, and 565, are crucial to the function of the VP2 capsid [2]. The dN - dS at sites 378, 383, 436, and 565 are 0.42, 0.4, 0.77, and 0.33, respectively, which indicates that all four residues are under positive selection.

### Molecular analysis of the VP2 capsid and amino acid mutations in important residues

**Figure 3:**
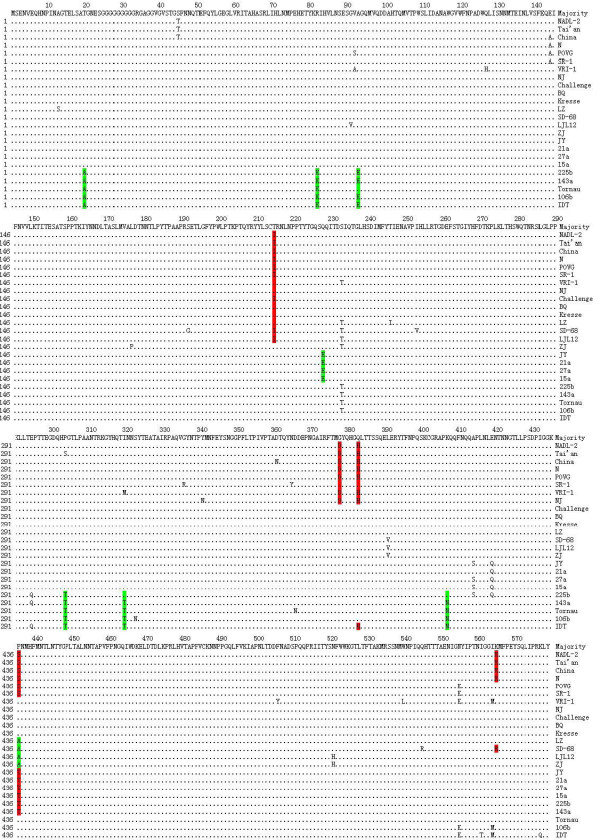
**Sequence alignment of the deduced amino acid sequences of the VP2 gene**. Important amino acid sites in the VP2 capsid are highlighted in different colors.

To find the molecular characteristics of the VP2 protein, the amino acid sequence alignments of the deduced VP2 coding sequences were constructed with representative strains aligned against the conserved sequence of all VP2 proteins. The alignment results of the complete VP2 proteins are presented in Figure 3. A number of polymorphic sites could be found along the VP2 amino acid sequence. Some mutations among the amino acids of the VP2 protein may distinguish different clades. For instance, Groups II and III viruses have a specific change at P436A and Q228E, respectively, whereas T20A, R82K, A93E, P304T, I320T, and K407N are unique to Group IV viruses.

In spite of the high levels of identity among the different parvovirus nucleotide sequences, parvovirus isolates can be distinguished by host range, cell tropism, and pathogenicity. In PPV, determinants for cell or tissue tropism, host range, and hemagglutination properties have been located within the capsid proteins. The biological significance of most of the sequence variations is unclear. However, some important amino acids in the VP2 protein have been identified by comparison of infectious clones of the NADL-2 and the Kresse strains [8,9], and by structure elucidation of the PPV capsids using X-ray crystallography [2]. For instance, the amino acids at positions 378, 383, and 436 are responsible for the allotropic determinant, as identified by the replacement of these residues with the Bgl II fragment in the infectious clone NADL-2. By mapping the key amino acid sites in the 3D structure of the capsid, some residues were found on the capsid surface. These residues are mapped to loop II for site 215, loops III and IV for sites 378 and 383, respectively, and to the C-terminal for site 565. Residue 436 is located on top of the three-fold spike within loop IV, which is the most accessible capsid structure and may be involved in tropism. Other residues, such as sites 314 and 509, are also important to the structural stability and biological function of the capsid because of their interaction with surface residues. As shown in Figure 3, the sequence difference mainly occurs at positions 215, 378, 383, 436, and 565. The Chinese viruses of Group I have S436 or P436 residues. Residue A436 is unique to the Group II Chinese viruses. The Group III viruses and most of Group IV have the unique residue T436. The isolates JY and LZ have residues at T215, G378, Q383, and K565. However, a significant difference was found in A436 of the LZ strain and T436 of the JY strain.

### Sequence diversity of NS1 and VP2 protein

**Figure 4:**
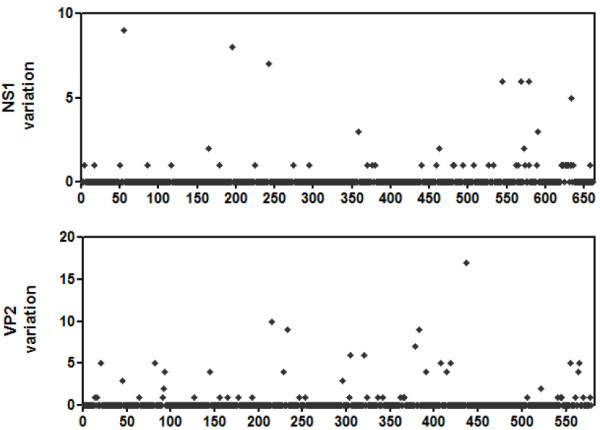
**Distribution of amino acid differences throughout the NS1 and VP2 proteins**. Dots represent the numbers of sequences differing from the consensus at each position. Numbers on the horizontal axis represent amino acid position

The distribution of genetic diversity across the NS1 and VP2 proteins was investigated for 23 NS1 sequences and 25 VP2 sequences included in the previous phylogenetic analysis (Figure 4). The polymorphic diversity of the VP2 protein sequences was greater than that of NS1 protein sequences. The NS1 protein sequences contain 50/662 (7.55%) polymorphic amino acid positions and have an overall amino acid diversity of 0.15 ± 0.03, whereas the VP2 protein sequences contain 48/579 (8.29%) polymorphic amino acid positions and have an overall amino acid diversity of 0.26 ± 0.05.

## Discussion

PPV is found in almost all pig-breeding countries. The PPV genome exhibits high genetic variation. Genetic analysis of the VP2 PPV gene in Brazilian isolates showed that two virus lineages existed in Brazilian swine populations [10]. Germany has a long PPV infection history. Hence, the genetic variability of German PPV isolates is complicated. Phylogenetic analysis of the full-length VP1 nucleotide sequences of German isolates revealed two co-circulating clusters [11]. A further infection experiment showed that viruses of these two clusters are immunologically different, and indicated that the variation in PPV is not only at the gene level, but also at the antigenic level. Genome recombination in PPV has also been reported [12]. Conflicting phylogenetic histories of the different PPV genome regions provide evidence for recombination within these regions.

Various PPV strains have been isolated in field samples from China [6,7]. In the phylogenetic tree, the early Chinese PPV isolates, such as PPV strains N, China, and SR-1, are mainly located in Group I. Almost all later Chinese PPV isolates are Group II viruses, which are composed of Chinese strains, such as PPV strains ZJ, NJ-2, and nanjing200801. The LZ isolate also belongs to group II in the NS1 and VP2 phylogenetic trees. JY, a Chinese PPV strain, falls under Group III, which is composed of German PPV isolates. This is the first isolation of a German-like PPV from field samples in China. This provides new molecular data on PPV strains in China, which may be used for etiological studies.

Considering the numerous PPV strains already sequenced, this study attempted to determine the genetic evolution of the PPV genome based on the selective pressures on the NS1 and VP2 genes. These PPV genes are under purifying selection and positive selection, respectively, as the average dN - dS for NS1 and VP2 genes are negative and positive, respectively. This suggests strong selection for the nonstructural proteins, which are less tolerant of amino acid alterations. The NS1 gene is more conserved than the VP2 gene. Hence, the NS1 gene may be regarded as the target gene for PPV detection. In contrast, the VP2 gene is inclined to mutate, especially in key points of the VP2 capsid, such as sites 378, 383, 436, and 565. These sites are all under positive selection, which indicates that the amino acid changes in these sites may be favorable for the survival of PPV.

The sequence lengths of the VP2 codon vary among different strains. The attenuated strain NADL-2 has a 127 bp repeat, and it is considered to be distinct from the virulent isolates [8]. However, the repeat phenomenon was also found in the two virulent Chinese strains, China and SR-1. Hence, determining the virulence of a field strain based solely on the length of the repeat sequence is difficult. The JY strain lacks the 127 bp repeat, similar to the field strains Kresse, 15a, Tornau, and 106b. The LZ strain, however, partially lacks nucleotides in the repeat region. Analogously, the lymphotropic variant of MVM, MVMi, also lacks a 65 bp repeat [13]. The tandem repeat might be important for the replication of the viral genome because an infectious clone of MVMp, which lacks one copy of the repeat sequence, replicates at approximately 10% of wild viral levels in A9 cells. In addition, the repeats are very AT-rich (77%) and may have a negative effect on the stability of the transcripts [14].

## Conclusions

The present study described the phylogenetic relationships, evolution, and genetic diversity of PPV strains isolated in China based on the NS1 and VP2 genes. A Group III virus from China was isolated for the first time. This study provides information for surveillance, prevention, and control strategies for PPV infections in China.

## Methods

### Field samples

From May 2009 to February 2010, swine in Gansu Province suffered from reproductive failure. Clinical samples, including lungs, kidneys, livers, and lymph nodes from pigs suffering from SMEDI (stillbirth, mummification, embryonic death, and infertility) were collected. Tissues samples from the pigs were homogenized for DNA extraction or virus isolation, and stored at -70°C. The sampling method was conducted in accordance with the guidelines on animal experimentation of Chinese academy of agricultural sciences (CAAS).

### Amplification of the of PPV whole sequences

The viral genome was directly extracted from the homogenized tissues using the EZNA Tissue DNA kit (OMEGA, USA).

To understand the genetic characteristics of the newly isolated PPV strains, five pairs of primers were designed according to the conservative region of the whole sequence of PPV (Table 1). The amplified regions cover the whole genome of PPV with the exception of two terminal palindromic structures. The PCR reactions contained 4 μl extracted DNA, 1 μl primer pairs, 3 mM dNTPs (TaKaRa, Dalian, China), 5 μl 10 × LA Taq buffer, and 0.5 U LA Taq polymerase (TaKaRa, Dalian, China) in a total volume of 50 μl. The PCR amplification was initiated by a pre-denaturation phage at 95°C for 5 min, followed by 30 cycles of denaturation at 94°C for 1 min, annealing at temperatures ranging from 54 to 59°C (Table 1) for 30 s, and an extension at 72°C for 30-100 s (depending on the amplified fragment length). Subsequently, the PCR products were ligated into a pMD18-T vector (TaKaRa, Dalian, China) after gel extraction, and were used to transform *Escherichia coli *DH-5α-competent cells (TaKaRa, Dalian, China). The positive bacterial suspensions were sent to Invitrogen Corporation (Shanghai, China) for sequencing. The sequence assembly was carried out using the SeqMan program of the DNASTAR Software (Madison, WI).

**Table 1:** Primers used in the amplification of the PPV genome.

Primer pairs	Location ^a^	Sequences (5'-3')	Annealing temperature (°C)	Amplicon length (bp)
A1	244-1497	CACTTCGCTCCAGAGACACAGCTA	58	1254
		TGTTGATGCTGGCCCATGAAATAG		
A2	1388-2356	TCAGCATGCACAATTGGAACTACA	56	969
		GTTTTATATGTATGCCCACCACCC		
A3	2214-3903	GGAAATAGAAACCGACATAAGAGC	55	1690
		TTATATTGTGTGTCTGCTGTTGGT		
A4	3796-4456	AATTAGGCCAGCTCAGGTAGGATA	59	661
		TGTTGTTGTGTGTTGTTGAATAGG		
A5	4239-4854	GACTACATGTTACAGCTCCATTTG	54	489-616 b
		ATAGTAAACACATGAGAGCTTGTT		

^a ^The position of the primer pairs are based on the entire genome sequence of the PPV NADL-2 strain [GenBank: NC_001718].

^b ^The PCR products between 489-616 bp were all regarded as positive samples and sequenced, as a fragment deletion of 127 bp or less may occur in the amplification region of the A5 primer pair.

### Sequence analysis

Multiple sequence alignment was carried out using CLUSTAL W of the MegAlign program (DNASTAR), and the unrooted phylogenetic trees of NS1 and VP2 gene were generated by the neighbor-joining method using MEGA 4.1 software http://www.megasoftware.net/mega4/mega41.html. Bootstrap values were calculated based on 1,000 replicates. Aside from the complete sequences of the two new Chinese strains JY and LZ, several genomic sequences were available from GenBank. All NS1 and VP2 genes available from GenBank were aligned to build the phylogenetic tree. The sequences of the VP2 proteins were also aligned to locate residue alterations. The virus isolates included 3 American strains, 8 Germany strains, 21 Chinese strains, 1 UK strain, and 1 South Korean strain (Table 2).

**Table 2:** Details of the PPV isolates used in this study.

Isolate	GenBank accession no.	Origin	Dataset	Reference
NADL-2	NC 001718	USA	NS1&VP2	[9]
Kresse	U44978	USA	NS1&VP2	[8]
POVG	D00623	USA	NS1&VP2	[17]
Challenge	AY644866	UK	NS1&VP2	[11]
VRI-1	AY390557	South Korea	NS1&VP2	Unpublished
106b	AY684870	Germany	VP2	[11]
143a	AY684867	Germany	VP2	[11]
15a	AY684865	Germany	VP2	[11]
21a	AY684868	Germany	VP2	[11]
225b	AY684864	Germany	VP2	[11]
27a	AY684871	Germany	VP2	[11]
IDT	AY684872	Germany	NS1&VP2	[11]
Tornau	AY684869	Germany	NS1&VP2	[11]
BQ	EU790641	China	NS1&VP2	[7]
ZJ	EU790642	China	NS1&VP2	[7]
China	AY553318	China	NS1&VP2	[6]
HN-Z1	AY789533	China	NS1	Unpublished
HN-Z3	AY789534	China	NS1	Unpublished
LJL12	DQ464345	China	VP2	Unpublished
N	EF212027	China	VP2	Unpublished
Nanjing-1	AY739664	China	NS1	Unpublished
Nanjing200801	FJ822038	China	NS1	Unpublished
Nanjing200802	FJ822039	China	NS1	Unpublished
NJ	AY686601	China	NS1	Unpublished
NJ	AY686602	China	VP2	Unpublished
NJ-2	AY789532	China	NS1	Unpublished
S-1	EU707335	China	NS1	Unpublished
SD-68	AY502114	China	NS1	Unpublished
SD-68	AY502115	China	VP2	Unpublished
SR-1	DQ675456	China	NS1/VP2	Unpublished
Tai'an	FJ853420	China	NS1	Unpublished
Tai'an	FJ853421	China	VP2	Unpublished
JY	HM627652	China	NS1&VP2	This study
LZ	HM627653	China	NS1&VP2	This study

The selective pressures on the PPV genome were assessed by calculating the differences between the non-synonymous (dN) and synonymous (dS) rates (dN - dS) for the NS1 and VP2 genes. The dN and dS rates were calculated using the SNAP web utility http://hcv.lanl.gov/content/sequence/SNAP/SNAP.html. SNAP calculates dN and dS rates for codon-aligned nucleotide sequences [15]. The ratios dN - dS > 0, dN - dS = 0, and dN - dS < 0 signify positive selection (adaptive molecular evolution), neutral mutations, and negative selection (purifying selection), respectively [16].

## Competing interests

The authors declare that they have no competing interests.

## Authors' contributions

ZJL and ZXL designed the experiments; XFH, PS, YFF, YMC, PHL, XWB, HFB, BXX, YLC, and DL performed the experiments and analyzed the data; XFH and ZJL wrote the paper. All authors read and approved the final manuscript.
